# How Is Science Teacher Job Satisfaction Influenced by Their Professional Collaboration? Evidence from Pisa 2015 Data

**DOI:** 10.3390/ijerph20021137

**Published:** 2023-01-09

**Authors:** Xinping Zhang, Xiaoxia Cheng, Yajing Wang

**Affiliations:** School of Educational Science, Nanjing Normal University, Ninghai Street, Nanjing 210097, China

**Keywords:** job satisfaction, science teacher collaboration, instructional self-efficacy, PISA

## Abstract

Due to the challenging nature of teaching and learning in the 21st century, educators must assume additional roles in schools to meet the expectations of students, parents, and communities. Studies in general have focused on all teachers as a group. The PISA 2015 assessment and analysis framework indicates that the focus of the current round of assessment is on science literacy. Therefore, science teacher professional collaboration, teaching self-efficacy, and teacher job satisfaction were also the focus of its measurement. In this study, 1039 science teachers from Hong Kong participated. Through literature review analysis, this study concluded that (a) teacher professional collaboration and teaching self-efficacy have a positive effect on job satisfaction; (b) teacher professional collaboration has a positive effect on teaching self-efficacy, and (c) teaching self-efficacy has a mediating role in teacher professional collaboration and teacher job satisfaction. A mediation model was developed to test this hypothesis. Data were analyzed using structural equation modeling (SEM). The results of the study confirmed our hypothesis. In addition, we examined the applicability of the model using multi-group SEM mode, and the results demonstrated that the effect of professional collaboration on job satisfaction among science teachers in Hong Kong, China did not differ by gender.

## 1. Introduction

The frequent resignation of teachers has become a common phenomenon recently. Researchers are concerned about this situation. As an example, Parker et al. [1] indicate that in the United States, teachers were most likely to quit within the first three years of employment. According to statistics, there is an attrition rate of up to 45 percent in the organization. This is arguably astonishing. One might therefore reflect on the need to consider what catalyzes such a high attrition rate. The same situation also exists in China [2]. It is well known that teachers are one of the most important resources for school development, and ensuring teacher development and stability is crucial to ensuring that the quality of education and teaching is improved as well as an important support for achieving quality and balanced education and sustainable development. There is no doubt that teachers, as the main implementers and participants in the education and teaching process, will play a direct role in influencing a student’s development in a positive way. Therefore, teacher job satisfaction is one of the most important indicators of teachers’ professional well-being, and an important basis for measuring the effectiveness of school management. On the other one hand, over the long term, teachers’ isolation negatively impacts their teaching self-efficacy and their ability to perform well at work [3,4,5,6]. Fortunately, teacher collaboration can mitigate the negative effects of teacher isolation [7,8].

In recent years, teacher-professional collaboration and teaching self-efficacy have received significant attention in academic research. Various empirical studies were conducted in this field [9,10,11,12]. Teacher professional collaboration is reported to be positively correlated with job satisfaction [13,14,15]. As for teaching self-efficacy, research has demonstrated that teachers who have higher teaching self-efficacy tend to obtain higher confidence in, and greater satisfaction with, their teaching [16,17]. Teachers would feel satisfied when they have higher teaching self-efficacy and peer collaboration in teaching. Meanwhile, teacher professional collaboration can reduce isolation and improve teaching self-efficacy. This indicates that teacher professional collaboration has an indirect effect on job satisfaction through teaching self-efficacy.

Science and technology are extremely important to a country and are related to the future and destiny of a country. PISA 2015 focused on investigating students’ science literacy and also focused on science teachers as a group [18]. On the basis of the literature review which was conducted, it can be said that most of studies are intended for a large group of teachers. On the subject of science teachers, there has been a dearth of research that focuses specifically on the job satisfaction of science teachers. Therefore, in this study, we analyze 1039 teachers’ job satisfaction levels in Hong Kong, China, based on data collected from the PISA 2015 teacher survey. Our research goal is to explore how science teachers’ professional collaboration affects their job satisfaction.

## 2. Literature Review

### 2.1. The Impact of Teacher Collaboration on Their Job Satisfaction

The term “collaboration” refers to the behavior of group members who move in tandem to achieve a common goal as a group. Based on the perspective of social interaction theory, cooperation among teachers in the school field can be regarded as an interpersonal interaction in a special social environment. The autonomy and independence of teachers’ work makes them want to carry out effective cooperation with others; share knowledge; exchange and interact in the cooperation; and promote the improvement and development of individual abilities. Research suggests that teachers’ collaborative professional development and teaching practices positively impact student learning, such as classroom observations and participation in collaborative teaching, which contribute to student academic performance [19]. According to an OECD study entitled Supporting Teacher Professionalism, a significant correlation was found between teacher development cooperation and both teaching effectiveness and job satisfaction [20]. In spite of this, there is still insufficient evidence to support the claim that collaborative teaching practices on teacher job satisfaction [21]. Several studies suggest that teacher collaborative teaching practices, including collaborative activities or learning communities about teaching and learning, facilitate the systematic sharing of experiences and expertise within the school community, thereby contributing to teacher job satisfaction [22].

It is generally accepted that teacher job satisfaction can be defined as a result of the relationship between a teacher’s expectations and how they perceive their job performance as a whole [23]. It is true that there have been a number of studies that have focused on the ways in which teachers can be more satisfied with their jobs in the educational setting [24]. DuFour et al. [25] theorized the positive impacts of a Professional Learning Community(PLC) and teacher collaboration on teacher job satisfaction. Banerjee et al. [26] indicate that high levels of teacher cooperation are associated with a significant positive impact on school performance, and teacher cooperation can improve student achievement. A study conducted in 2013 suggests that teachers often discuss teaching theories, teaching methods, teaching, and learning, which improves both job satisfaction and teaching self-efficacy [27]. Torres [13] used the TALIS 2013 data to analyze the prior relationship between teacher professional collaboration and their satisfaction with the work they do.

Although there was some research on teacher job satisfaction, the results have yielded some positive results. However, only a small number of studies have specifically focused on the relationship between science teacher job satisfaction and professional collaboration. According to OECD research, there appears to be a positive correlation between students’ achievement in science literacy and the degree of cooperation between teachers of science. In PISA 2015 study, the authors found that only professional cooperation between science teachers was significantly correlated with student achievement when controlling for factors such as student and school socioeconomic status. OECD’s result shows that students in Slovenia score 36 points higher in science when principals report that their teachers cooperate professionally, compared to those who do not report [28]. There is an enormous gap between the two of them. With this section of the literature review, we conjecture that science teacher professional collaboration positively influences science teachers’ job satisfaction.

### 2.2. Influence of Teacher Collaboration on Teachers’ Instructional Self-Efficacy

Teaching self-efficacy is the level of confidence a teacher has in his or her ability to guide and motivate students so that they will be able to achieve a certain degree of success [29]. It was shown in several studies that professional collaboration has significant positive associations with teacher self-efficacy [9], through group cooperation, and the formation of a friendly partnership can promote teachers ‘knowledge, information, and emotion sharing, and then improve teachers’ teaching efficiency [30]. Moreover, Chong and Kong [31] also suggest that teachers’ self-efficacy is enhanced by systematic support of their instructional development. It was found in a study by Goddard and Kim [5] that higher self-efficacy among teachers of primary schools was more positively correlated with formal collaborative efforts, informal collaborative activities, and instructional policy collaborations. Moolenaar et al. [32] found that increased confidence and self-efficacy can be attributed to the benefits of collaboration. However, there are definitional inconsistencies that complicate the evaluation of what is likely to make teacher collaboration effective or ineffective, because many studies have also come to the conclusion that incorporating teacher collaboration has negative outcomes [33]. Therefore, it is imperative that we examine the relationship between science teacher professional collaboration and teaching self-efficacy based on professional data such as OECD data.

### 2.3. Impact of Teacher Instructional Self-Efficacy on Their Job Satisfaction

The self-efficacy of teachers can be defined as their perception of their ability to influence the learning outcomes of their students in their classrooms [34]. Teacher teaching self-efficacy is not only the main component of teacher professional quality and teaching belief, but also one of the most important indicators that can be used to measure teachers’ teaching effect and education quality. Researchers use teachers to successfully influence students’ learning and teaching in their teaching activities. The ability perception and belief of effect are defined as teaching efficacy [35]. Job satisfaction may be related to a number of factors, such as those associated with the quality of a teacher’s teaching, student management, and the way the classroom is managed [36,37]. Liu, Bellibaş, and Gümüş [11] discovered that there is a positive correlation between teacher self-efficacy and job satisfaction. Moreover, self-efficacy is not only positively correlated to teaching belief, organizational commitment, and job satisfaction, but also demonstrates an important relationship between the teacher teaching belief and the learning outcome of student development, and plays a positive role in promoting effective teaching and school efficiency [38,39].

Teacher job satisfaction can be viewed as a very complex psychological system. There have been remarkable results regarding teacher job satisfaction, burnout, performance, and organizational commitment have achieved remarkable results, but there are still some shortcomings in studies regarding the factors influencing teacher job satisfaction. There are more studies about the correlation or regression between teachers’ job satisfaction and other variables, but the paths of action between these variables are not very clear, and there is a lack of specific recommendations for the design and implementation of relevant intervention programs in the future, and there are fewer studies on science teachers’ job satisfaction. Therefore, based on related studies, an empirical study was conducted with secondary school science teachers to provide empirical references for future education departments to develop relevant systems and measures aimed at improving science teacher job satisfaction. We extended this work by investigating how science teacher job satisfaction is influenced by professional collaboration.

### 2.4. Theoretical Framework

**Figure 1:**
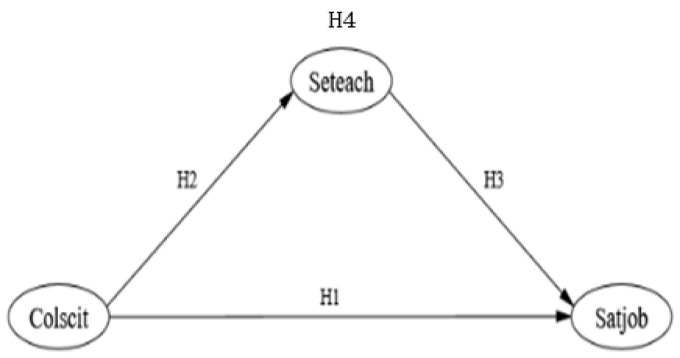
A hypothetical model relating science teacher job satisfaction, teacher collaboration, and instructional self-efficacy.

Literature review shows that teacher professional collaboration is significantly correlated with teacher job satisfaction and teaching self-efficacy, and teaching self-efficacy is also significantly correlated with teacher job satisfaction. Therefore, our research team developed a theoretical model (Figure 1). It can be summarized in the following way:

**H1**:  *Teacher collaboration can influence their job satisfaction.*

**H2**:  *Teacher collaboration can influence their instructional self-efficacy.*

**H3**:  *Teaching self-efficacy can influence their job satisfaction.*

**H4**:  *Teaching self-efficacy mediates relationships between teacher professional collaboration and teacher job satisfaction.*

## 3. Research Methodology

### 3.1. General Background

As part of our study, we aim to solve quantitative research problems and attempt to answer the following questions: what is the relationship between science teacher job satisfaction and teacher professional collaboration? How does teacher professional cooperation affect teacher job satisfaction in science? To ensure that the research is rigorous, it is necessary to elaborate on the following aspects: the research model, the sample, the source of data, how the data were collected, and how the data were analyzed.

### 3.2. Sample

PISA is an acronym for Program for International Student Assessment, and only 15-year-old students can take the test. It is estimated that applying their knowledge and skills in science, reading, and mathematics in a real-life situation is assessed through the PISA test. Countries can add a questionnaire for teachers in order to have a more comprehensive understanding of the learning environment of students [18]. The questionnaire is available in two versions, one for science teachers and one for other teachers. It takes about 30 min to complete the questionnaire if you are a science teacher. The 2015 PISA dataset was chosen for the present study because the primary test focus was on science literacy compared to reading literacy in PISA 2018 [40]. The data for this study were taken from the PISA 2015, which can be found on the official website of the OECD. During the evaluation of the 2015 Science teacher questionnaire, strict adherence to the relevant theoretical framework was emphasized as part of the project evaluation [18]. As a professional project team, OECD has established strict testing procedures and established quality monitoring systems in addition to data management procedures in order to ensure that scientific data is reliable [41]. The Cronbach’s alpha of the three constructs were found to be within a range of values from 0.732 to 0.903 [41].

We have downloaded the PISA 2015 technical report, codebook, and teacher questionnaire data file (SPSS version) from the official website of PISA China [42]. In KeyQuest, teachers were classified into Population 4 (science teachers) and Population 5 (nonscience teachers), and the country code for Hong Kong, China is 344 [41]. SPSS has a function for selecting cases [43], we selected teachers from Hong Kong (China) as the participants, denoted as Hong Kong (CNTRYID: 344) in the official datasets. Then, according to the PISA 2015 technical report [41] and the content of our research, we extracted the participants’ teacher IDs as well as the 18 observed variables of each participant, including gender. Using these data [42], this study was completed. A total sample size of 1039 science teachers was obtained. Missing values were removed, and the final sample number is shown in Table 1. The percentage of males was 66.98% and 33.02% of females. We think that this is a fairly reasonable percentage because reflecting reality. In general subjects, the percentage of females is much greater than the percentage of male teachers.

### 3.3. Instrument and Procedures

#### 3.3.1. Teacher Collaboration

Using a Likert scale of four points, science teachers’ collaboration (Colscit) was assessed. The purpose of this survey is to evaluate to what extent teachers engage in professional cooperative practices and activities to achieve common educational goals, ranging from “strongly disagree “to “strongly agree” regarding different aspects of cooperation. Scores from the instrument are reliable and valid [41]. In Appendix A (TC031), you will find a listing of the scale items in this construct.

#### 3.3.2. Teacher Job Satisfaction

A set of questions were asked about how satisfied they were with the job of science teachers. As indicated by the Likert scale, there are four levels of agreement: “strongly agree”, “agree”, “disagree”, and “strongly disagree”. According to the PISA2015 technical document, science teacher job satisfaction can be divided into two dimensions: job satisfaction with the profession (4 items) and job satisfaction with the work environment (4 items). The Cronbach’s alpha of the two dimensions is 0.697 and 0.805, respectively, indicating that the job satisfaction with the work environment variable has high reliability. This study used partial items to examine the factors that are influencing teacher job satisfaction (5 items). In addition, gender was included in this study to fully reflect the differences among different teachers. Detailed information about the scale items for this construct can be found in Appendix A (TC026).

#### 3.3.3. Teacher Instructional Self-Efficacy

A set of questions were asked about what level of job satisfaction is experienced by science teachers. In response to the Likert scale, there are four levels of agreement: “not at all”, “very little”, “to some extent”, and “to a great extent”. Self-efficacy of teachers in delivering science content in everyday classrooms (Seteach) such as the use of experiments to teach science content in everyday classrooms. The reliability coefficients of the dimensions are 0.732. Appendix A (TC033) provides information on the items on the scale for this latent variable.

### 3.4. Data Analysis

There were three steps in the analysis process following the steps of the SEM model analysis and the operation points of AMOS. In the first stage, the data were analyzed using descriptive statistics. Second, SEM analysis was performed. The third step compared the model equivalence of male and female science teachers through the multi-group SEM mode.

Initially, descriptive statistics were used with SPSS 23 as part of the first stage of the study (see Table 2). In addition to this, we also focused our attention on the correlations, which were key to the subsequent SEM analysis. In the SEM analysis, 17 of the items included in the assessment were used as the observed variables, and correlation coefficients were calculated for each latent variable to determine whether these variables have a direct relationship. Please refer to Table 2 and Table 3 for more information.

It was then that AMOS 24 was tasked with performing the second phase of the analysis. Our decision to use an SEM model and AMOS for the analysis of the data was based on the following considerations, which led us to make our decision. SEM offers the possibility to use a limited number of variables in order to investigate a complex construct in the simplest possible way. It is possible to conduct this type of analysis very easily and use SEM to model and statistically test the relationships between multiple variables, a better method than using theoretical models to confirm (or disprove) these models. A key feature of this software is the ability to drag and drop variables from the SPSS data set into the model, analyze multiple models at once, and perform canonical searches to find one that is more accurate [44]. As a first step, we conducted an SEM analysis to test the hypothesis. In particular, we examined the results of the computational analysis of model fitness indicators (see Table 4). And through the setting of AMOS-related parameters, the effect sizes of the mediation model were analyzed using the Boostrap method to obtain the path coefficients between the three variables and to determine whether the mediation effects were significant. Table 5 and Table 6 are provided for your information.

In the third stage, we analyzed whether the study model differed for male and female science teachers using the multi-group SEM mode function that comes with AMOS. As a result, we are presenting the results in Table 7, Table 8 and Table 9.

## 4. Research Results

### 4.1. Descriptive and Correlation Results

In Table 2, there is a range of values for skewness between −0.011 and −0.305, and a range of values for kurtosis between −0.231 and 2.632. Taking into consideration the criteria that are used to approximate normality suggested by Kline [45], it appears that the observed variables follow a relatively normal distribution.

According to Table 3, we were able to perform the analysis by taking the item means of each construct through SPSS software. It has been demonstrated that science teacher professional collaboration has a significant positive correlation with teaching self-efficacy (r = 0.316, *p* < 0.01) and teacher job satisfaction (r = 0.316, *p* < 0.01). Teacher self-efficacy was significantly correlated with teacher job satisfaction (r = 0.227, *p* < 0.01). It is important to note that these indicators are a solid foundation for SEM analysis. As a result of the correlation analysis that has been conducted, there is clearly a relationship between these three variables at first glance. To further explore the specific path of the relationship between them, the next step will be to develop a structural equation model using these variables for analysis.

### 4.2. Structural Models

**Figure 2:**
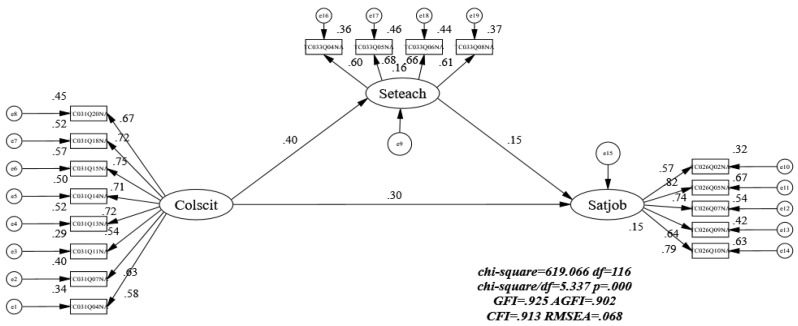
The Result of Mediation Model for the Science Teacher.

In this study, structural equation models (SEMs) are employed in order to test hypotheses and develop a model with intuitive explanations for the association between science teachers’ professional collaboration, their teaching self-efficacy, and their feelings of job satisfaction in the classroom. For the estimation of the model, we chose the maximum likelihood method (ML), a technique that is commonly used to deal with data sets derived from large-scale surveys, such as the TALIS and PISA, which use structural equation modeling (SEM) [46,47,48]. Based on the results in descriptive statistics (see Table 2), it is reasonable to assume that the data in this study are normally distributed. After testing and modifying the model, a final model was constructed (see Figure 2). Using this model, we are able to understand how science teacher professional collaboration, teaching self-efficacy, and job satisfaction interact with each other.

It was determined that the most accurate model for this study is the one shown in Figure 2, as it is the most accurate model for the data in this study. Table 4 summarizes the metrics of model fit and their calculations [44,45]. The model fit indices indicated that the structural model had an acceptable fit (χ2 = 619.066, df = 116, RMSEA = 0.068, CFI = 0.913, TLI = 0.898). As the sample size increases (typically above 200), the χ2 statistic tends to reach a significant level of significance [44]. It is found that χ2 of the model is influenced by the size of the sample. In this study, we examined a large sample of over 900 science teachers, leading to a significant increase in χ2 and a significant *p*-value. In order to determine if the hypothesized model would be acceptable, it is reasonable to consider most of the indicators rather than just the *p*-values. As a result, Figure 2 was selected as the final model that best matched the data and thus was accepted as the final model. The direct effects are presented in Table 5 and Figure 2. The results indicated that teacher professional collaboration positively and significantly predicted job satisfaction (β = 0.303, *p* < 0.001) and teaching self-efficacy (β = 0.402, *p* < 0.001). Meanwhile, teaching self-efficacy had a significant positive impact on job satisfaction (β = 0.154, *p* < 0.001). The research hypotheses (H1, H2, and H3) have been verified.

### 4.3. Mediation Analyses of The Science Teacher

In order to verify hypothesis 4, we calculated the indirect effects of teacher professional collaboration on job satisfaction through the mediating variable of teaching self-efficacy. This study will allow us to understand the relationship between science teachers and these three constructs in a broader sense. As suggested by Hayes [49], the indirect effect is significant if zero is not between the lower and upper bound in the 95 % confidence interval (CI). According to Table 6, science teaching self-efficacy mediate relationships between teacher professional collaboration and job satisfaction. Meanwhile, teaching self-efficacy, although it played an indirect role (the results were significant). However its effect value (β = 0.075) was, much smaller than the direct effect (β = 0.364) of professional collaboration on job satisfaction.

### 4.4. Multi-Group SEM Model by Gender

Further analysis of the data was carried out with the aim of determining whether gender-related models could be applied. We selected gender as a categorical variable to examine the model fitness for male science teachers and female science teachers. Using the model fitness indicators, Table 7 indicates whether or not there is a significant difference between the two models. In the second step, we calculated the path coefficients of the models and the results are presented in Table 8. Finally, we checked the significance of the differences, and the results are presented in Table 9. In this respect, it can be considered as a general model that can be applied to many types of groups.

## 5. Discussion

### 5.1. The Impact of Science Teacher Collaboration on Job Satisfaction

The results of the study have supported the hypothesis as a consequence of the results. It is possible to accept the hypothesized model as the final model explaining the relationship between science teacher professional collaboration, teaching self-efficacy, and job satisfaction among science teachers in Hong Kong. According to this model, there is a true association between the three latent variables. According to Table 4, science teachers’ professional collaboration directly influenced teacher teaching efficacy (β = 0.402) and teacher job satisfaction (β = 0.303). Teacher teaching efficacy also positively influenced teacher job satisfaction (β = 0.154). All three paths were significant (*p* < 0.001). We can further conclude that teacher professional collaboration is more important than teacher teaching efficacy in terms of science teacher job satisfaction. Science teacher job satisfaction is indirectly influenced by teacher professional collaboration mediated by teaching self-efficacy. If we want to raise the level of job satisfaction of science teachers, we will be able to increase teacher collaboration and teaching effectiveness. As a result of higher levels of confidence in teachers, it was found that students perform better and teachers are more content with their work [32,50].

### 5.2. The Mediation of Seteach between Science Teacher Collaboration and Job Satisfaction

Science teacher job satisfaction and professional collaboration are positively correlated. As a result of this study, a second finding was made. By examining Table 6, we concluded that science teacher teaching self-efficacy partially mediates the relationship between their professional collaboration and their job satisfaction. The present research has demonstrated that teaching self-efficacy is an important factor that contributes to the level of professional collaboration and satisfaction among science teachers. Those teachers who valued professional collaboration to gain feedback from others about their subject matter knowledge were able to achieve higher levels of teaching self-efficacy and job satisfaction when they refined their subject matter literacy and teaching skills. By reading professional books and journals, individual science teachers can acquire subject matter knowledge, but it is unlikely that they will acquire deep knowledge of the subject matter in a relatively short period of time. Conversely, teachers participate in a variety of training programs and workshops to discuss teachers’ subject knowledge, as well as collaborate to develop science education. In this way, teachers are able to gain a deeper understanding of the subject’s characteristics and appreciate its unique benefits. If teachers have a clearer understanding of the nature and nurturing value of discipline, they will reposition their role as teachers and become more satisfied with the teaching profession. Table 6 indicates that the direct effect of teacher professional collaboration on teacher job satisfaction (0.364) was greater than the indirect effect of teacher job satisfaction through teacher teaching efficacy (0.075). Yet, the significance of Bootstrap analysis showed that both its mediating and direct effects were significant. However, we still believe that science teacher professional collaboration is particularly significant for teacher job satisfaction compared to teaching efficacy. Nevertheless, this does not negate the positive impact of science teacher teaching self-efficacy on teacher job satisfaction.

Through multi-group SEM mode analysis, it was found that the effect of science teacher professional collaboration on teaching efficacy was higher for females than for males (females = 0.451, males = 0.382). The effect of professional collaboration on teacher job satisfaction was smaller for females than for males (females = 0.221, and males = 0.340). The effect of teaching self-efficacy on teacher job satisfaction was less for females than for males (females = 0.139, and males = 0.165). There is no significant difference in the effect of professional collaboration on job satisfaction among science teachers in Hong Kong, China, by gender. This finding is consistent with the actual situation. According to a literature review, there was a large amount of research demonstrating a positive correlation between teacher professional development and teacher job satisfaction [13,20,25]. Although we selected science teachers in Hong Kong, China, for this study, we even ventured to guess that this model could be applied to different cultural background regions as well as different types of teacher groups. Thus, teacher professional collaboration and teacher teaching efficacy are important for improving teacher job satisfaction. Of course, this conjecture needs to be proven by further analysis to demonstrate the usability of the model.

## 6. Conclusions and Implications

Research has shown that advocates for teacher collaboration tend to emphasize its potential benefits, which may include increased teacher job satisfaction, improved student achievement, and enhanced teacher confidence [51]. This study utilized structural equation modeling to examine the effects of science teacher professional collaboration on teaching self- efficacy and job satisfaction and to examine the mediating role of science teaching self-efficacy in this context. It was found by the findings of this study that not only did teacher professional collaboration directly and significantly affect teaching efficacy and job satisfaction, but teacher professional collaboration also affected teacher job satisfaction through the mediator of teacher teaching self-efficacy.

As a result of this study, it was found that professional collaboration among science teachers has a considerable impact on teaching self-efficacy, in line with previous research. For example, some researchers suggest that frequent organization of teachers’ sharing of teaching experiences and cooperative learning can promote teachers’ teaching efficacy. Science teacher job satisfaction was also significantly influenced by teacher professional collaboration. During the 1950s, Frederick Hertzberg proposed the “motivational-health factor theory” [52]. According to this theory, motivational factors are a group of factors that contribute to worker satisfaction, including a sense of accomplishment from the job, recognition of job performance, interest and challenge in the job itself, and a sense of responsibility in the position. By enhancing professional collaboration among teachers, teachers are more likely to solve complex educational and teaching problems together, feel satisfied with their work and gain a sense of accomplishment. It is beneficial for teachers to receive professional recognition from each other and helps teachers to increase their sense of responsibility through mutual learning and communication. It is through participation in professional collaboration activities that teachers enhance their professional competence and professionalism, improve the quality of their teaching, and receive professional recognition from others that contribute to the high level of satisfaction that teachers have as educators. The collaboration of novice teachers can be particularly beneficial for developing their confidence, thereby reducing brain drain among teachers by allowing them to learn from more experienced colleagues and recognizing that they are not the only ones facing particular challenges [53]. The Professional Learning Communities in Hong Kong, China, are an example of structured teacher collaboration. Through participating in PLCs, teachers feel more connected to their schools and colleagues [54]. Teachers are often organized to contribute to the development of national policies and guidelines, identify instructional objectives, collaborate to prepare and improve instruction, organize observation visits, provide feedback to colleagues, and organize activities for teachers outside of the classroom [55]. It is also noteworthy that there is a common practice among teachers in Japan known as “curriculum research” [56]. TALIS results further support the finding that there is a difference between the patterns of activities in different countries [57]. It is evident from all the above discussions that professional collaboration is important for science teachers in order to achieve success.

As a result of the findings of the empirical study, we would like to suggest three points. As a first step entails the establishment of a school institutional environment and a culture of teacher collaboration that is conducive to professional collaboration among science teachers. A friendly collaborative atmosphere among science teachers will enhance teaching self-efficacy, improving students’ science literacy as a result. Second, active professional collaboration activities will enhance teachers’ understanding and grasp of subject matter knowledge. The development of professional cooperation activities can not only enhance teachers’ sense of teaching efficacy, but also improve teachers’ sense of subject knowledge efficacy to a certain extent. According to TPACK theories [58], teachers in the 21st century need to have comprehensive scientific literacy, which is also applicable to science teachers. Another area of focus is the development of science teachers’ pre-service science knowledge and self-efficacy as teachers of science. A number of relevant studies have shown that teachers’ pre-service teaching efficacy can significantly affect their teaching satisfaction after work [58]. Therefore, it is especially important and feasible to pay attention to the development of science teachers’ pre-service general competence. Lastly, it is important to mention that professional development has a positive impact on the job satisfaction of science teachers. There is an increasing amount of work pressure being placed upon teachers due to the advent of the fourth industrial revolution. How to resolve this challenge is currently a hot topic of concern in countries around the world. Our study reveals that teacher professional collaboration and teacher teaching efficacy can improve the job satisfaction of science teachers. Our findings are expected to be taken seriously and adopted by relevant policy-making authorities.

This study has several limitations. First, the survey data from PISA 2015 is self-reported, so the relationship between teacher professional collaboration, teacher self-efficacy in teaching, and teacher job satisfaction in this study reflects the teachers’ perceptions rather than objective indicators. Therefore, future research could conduct some synthesis of the use of other methods, such as fieldwork, qualitative data, and experimental design. Secondly, this survey used a cross-sectional survey design, which means we cannot provide evidence to prove the causal relationship between these three variables. Therefore, future research should use longitudinal surveys or interview surveys to explore causal relationships. Thirdly, this study reveals the direct and indirect effects of professional collaboration among science teachers on job satisfaction through the mediating variable of science teachers’ teaching self-efficacy. However, China is vast and there are large regional differences, with significant differences in the development of schools in different regions. Therefore, the sample from Hong Kong in China from PISA 2015 used in this study may not produce results that are representative of the entire country. Therefore, future research should be conducted in other regions of China.

## Figures and Tables

**Table 1:** Participants are distributed according to their gender.

	Male	Female	Total
N	633	312	945
Percentage	66.98%	33.02%	100%

**Table 2:** The descriptive statistics of each variable observed (*n* = 945).

		Minimum	Maximum	Mean	S.D.	Skewness	Kurtosis
Colscit	TC031Q04NA	1	4	3.05	0.567	−0.305	1.314
	TC031Q07NA	1	4	2.98	0.637	−0.279	0.369
	TC031Q11NA	1	4	3.34	0.606	−0.538	0.457
	TC031Q13NA	1	4	2.87	0.653	−0.298	0.309
	TC031Q14NA	1	4	2.88	0.678	−0.424	0.463
	TC031Q15NA	1	4	3.11	0.556	−0.295	1.567
	TC031Q18NA	1	4	2.95	0.574	−0.405	1.282
	TC031Q20NA	1	4	2.97	0.565	−0.256	0.957
Seteach	TC033Q04NA	1	4	2.61	0.659	−0.021	−0.214
	TC033Q05NA	1	4	2.51	0.697	−0.011	−0.231
	TC033Q06NA	1	4	2.76	0.648	−0.345	0.283
	TC033Q08NA	1	4	2.88	0.620	−0.268	0.42
Satjob	TC026Q02NA	1	4	3.04	0.695	−0.448	0.313
	TC026Q05NA	1	4	2.93	0.655	−0.612	1.136
	TC026Q07NA	1	4	2.72	0.717	−0.411	0.132
	TC026Q09NA	1	4	3.07	0.537	−0.312	1.976
	TC026Q10NA	1	4	3.04	0.536	−0.502	2.632

**Table 3:** Descriptive statistics of each latent variable (*n* = 945).

Variable	Mean	S.D	Correlation Coefficient Pearson		
			1	2	3
Colscit	3.018	0.433	1		
Satjob	2.96	0.487	0.316 **	1	
Seteach	2.69	0.488	0.316 **	0.227 **	1

Note: ** *p* < 0.01.

**Table 4:** Indicators of model fitness and their computational results.

	P	GFI	AGFI	RMSEA	NFI	RFI	IFI	TLI	CFI
Results	0.000	0.925	0.902	0.068	0.896	0.878	0.914	0.898	0.913

**Table 5:** The weights of the regression model.

	Estimate (Unstandardised)	Estimate (Standardised)	S.E.	C.R.	*p*
Colscit—>Seteach	0.461	0.402	0.054	8.523	***
Colscit—>Satjob	0.364	0.303	0.055	6.671	***
Seteach—>Satjob	0.162	0.154	0.047	3.432	***

Note: *** *p* < 0.001.

**Table 6:** Mediation of effect of model.

	Point Estimation	Product of Coefficients		Bootstrapping			
				Bias-Corrected 95%CI		Percentile 95%CI	
		SE	Z	Lower	Upper	Lower	Upper
Indirect	0.075	0.025	3.000	0.03	0.131	0.028	0.128
Direct	0.364	0.064	5.688	0.248	0.503	0.241	0.494
Total	0.439	0.062	7.081	0.331	0.574	0.32	0.562

Note: Standardized estimation based on 5000 bootstrap samples.

**Table 7:** Results of indicators of multi-group SEM model fitness by gender.

	P	NFI	RFI	IFI	TLI	CFI	GFI	AGFI	RMSEA
Unconstrained	0.000	0.875	0.854	0.910	0.894	0.909	0.911	0.883	0.049
Measurement weights	0.000	0.874	0.860	0.910	0.900	0.910	0.910	0.888	0.048
Structural weights	0.000	0.873	0.861	0.910	0.902	0.910	0.910	0.889	0.047
Structural covariances	0.000	0.873	0.862	0.910	0.902	0.910	0.910	0.889	0.047
Structural residuals	0.000	0.873	0.863	0.910	0.903	0.910	0.909	0.890	0.047
Measurement residuals	0.000	0.869	0.868	0.909	0.908	0.909	0.907	0.894	0.046

**Table 8:** Effect of multi-group SEM model by gender.

	Female	Male
	Estimate (Standardised)	Estimate (Standardised)
Colscit—>Seteach	0.451	0.382
Colscit—>Satjob	0.221	0.34
Seteach—>Satjob	0.139	0.165

**Table 9:** Results of indicators of multi-group SEM mode invariance by gender.

	P	ΔNFI	ΔRFI	ΔIFI	ΔTLI	ΔCFI	ΔGFI	ΔAGFI
Measurement weights	0.655	−0.001	0.006	0.000	0.006	0.001	−0.001	0.005
Structural weights	0.647	−0.002	0.007	0.000	0.008	0.001	−0.001	0.006
Structural covariances	0.707	−0.002	0.008	0.000	0.008	0.001	−0.001	0.006
Structural residuals	0.640	−0.002	0.009	0.000	0.009	0.001	−0.002	0.007
Measurement residuals	0.477	−0.006	0.014	−0.001	0.014	0.000	−0.004	0.011

## Data Availability

Publicly available datasets were analyzed in this study. This data can be found here: https://www.oecd.org/pisa/data/2015database/ (accessed on 6 February 2022).

## Appendix A

**Table:**

Construct	Items	Item Coding
Colscit	We discuss the achievement requirements for science when setting tests.	TC031Q04NA
(TC031)	It is natural for us to cooperate on what homework to give to our students.	TC031Q07NA
	We discuss the criteria we use to grade written tests.	TC031Q11NA
	We exchange lesson plans and homework that cover a range of different levels of difficulty.	TC031Q13NA
	I prepare a selection of teaching units with my fellow science teachers.	TC031Q14NA
	We discuss ways to teach learning strategies and techniques to our students.	TC031Q15NA
	My fellow science teachers benefit from my specific skills and interests.	TC031Q18NA
	We discuss ways to better identify students’ individual strengths and weaknesses.	TC031Q20NA
Seteach	Design experiments and hands-on activities for inquiry-based learning	TC033Q04NA
(TC033)	Assign tailored tasks to the weakest as well as to the best students	TC033Q05NA
	Use a variety of assessment strategies	TC033Q06NA
	Facilitate a discussion among students on how to interpret experimental findings	TC033Q08NA
Satjob	I would recommend my school as a good place to work.	TC026Q02NA
(TC026)	I enjoy working at this school.	TC026Q05NA
	I would recommend my school as a good place to work.	TC026Q07NA
	I am satisfied with my performance in this school.	TC026Q09NA
	All in all, I am satisfied with my job.	TC026Q10NA
